# Angiotensin-2-Mediated Ca^2+^ Signaling in the Retinal Pigment Epithelium: Role of Angiotensin-Receptor- Associated-Protein and TRPV2 Channel

**DOI:** 10.1371/journal.pone.0049624

**Published:** 2012-11-20

**Authors:** Rene Barro-Soria, Julia Stindl, Claudia Müller, Renate Foeckler, Vladimir Todorov, Hayo Castrop, Olaf Strauß

**Affiliations:** 1 Experimental Ophthalmology, Eye Hospital, University Medical Center Regensburg, Regensburg, Germany; 2 Institute of Physiology, University of Regensburg, Regensburg, Germany; 3 Laboratory for Experimental Nephrology, Dresden University of Technology, Dresden, Germany; University of Florida, United States of America

## Abstract

Angiotensin II (AngII) receptor (ATR) is involved in pathologic local events such as neovascularisation and inflammation including in the brain and retina. The retinal pigment epithelium (RPE) expresses ATR in its AT1R form, angiotensin-receptor-associated protein (Atrap), and transient-receptor-potential channel-V2 (TRPV2). AT1R and Atrap co-localize to the basolateral membrane of the RPE, as shown by immunostaining. Stimulation of porcine RPE (pRPE) cells by AngII results in biphasic increases in intracellular free Ca^2+^inhibited by losartan. Xestospongin C (xest C) and U-73122, blockers of IP3R and PLC respectively, reduced AngII-evoked Ca^2+^response. RPE cells from Atrap^−/−^ mice showed smaller AngII-evoked Ca^2+^peak (by 22%) and loss of sustained Ca^2+^elevation compared to wild-type. The TRPV channel activator cannabidiol (CBD) at 15 µM stimulates intracellular Ca^2+^-rise suggesting that porcine RPE cells express TRPV2 channels. Further evidence supporting the functional expression of TRPV2 channels comes from experiments in which 100 µM SKF96365 (a TRPV channel inhibitor) reduced the cannabidiol-induced Ca^2+^-rise. Application of SKF96365 or reduction of TRPV2 expression by siRNA reduced the sustained phase of AngII-mediated Ca^2+^transients by 53%. Thus systemic AngII, an effector of the local renin-angiotensin system stimulates biphasic Ca^2+^transients in the RPE by releasing Ca^2+^from cytosolic IP3-dependent stores and activating ATR/Atrap and TRPV2 channels to generate a sustained Ca^2+^elevation.

## Introduction

The classical view of the Renin-Angiotensin System (RAS) as a systemic regulator of blood pressure has been extended, and a substantial number of studies have highlighted the importance of local RAS in a variety of extra-renal tissues, including adrenal glands [1], thymus [2] and recently in the eye [3], [4]. In the eye, angiotensin II type 1 receptors (AT1R) have been found in the retina, particularly in Müller cells, amacrine cells, photoreceptors, choroid and in the retinal pigment epithelium (RPE) [5]–[8]. Similarly, studies in rat retinal tissues also suggested local synthesis of both renin and angiotensin converting-enzyme (ACE) [9]. Along this line, Milenkovic et al. (2010) demonstrated that the RPE expresses renin and secretes it towards the retinal side. The presence of the most important RAS components in the retina implies a physiological function of RAS within the eye. However, despite the considerable evidence for local RAS in the retina, its exact role and its possible relationship with the systemic RAS remain poorly understood.

Angiotensin II (AngII) mediates its biological effects through the activation of AT1R and angiotensin II type 2 (AT2R) receptors. It is, however, through AT1R activation that AngII elicits most of its well known effects, including vasoconstriction, electrolyte homeostasis, fibrosis, inflammation and proliferation. In the eye, AT1R activation has been implicated in the pathogenesis of many ocular disorders such as diabetic retinopathy [10], [11], neovascularization in hypoxic-induced retinopathies [12]–[14] and age-related macular degeneration [15]–[17].

In addition, proteins that directly interact with AT1R have received considerable attention because of their crucial roles in the first steps in signal transduction. Among them, the angiotensin receptor-associated protein (Atrap) is the best described [18]. Based on the data published so far, it appears that Atrap functions as a negative modulator of AT1R in the renal tubules [19], [20].

The RPE plays an important role in maintaining retinal function by transporting nutrients, isomerizing all-trans to 11-cis retinal, and phagocytosing shed photoreceptor outer segments [21]. By its anatomical location, the RPE forms the interface between the retina and the blood supply from the choroid and represents a part of the blood-retinal barrier. This makes the RPE suitable to serve as a mediator for transfer of molecules and signals between the blood and the outer retina. The fact that AT1R is localized at the basolateral membrane [7], which faces the blood side of the epithelium, suggests that the activity of the systemic RAS is a part of that signaling. Interestingly, it has been shown that modulation of the systemic RAS (e.g. by systemic application of ACE inhibitors) changes neuronal activity within the retina, mainly of bipolar cells and amacrine cells as monitored by electroretinography [8], [22], [23]. Moreover, modulators of the systemic RAS alter renin expression in the RPE [7] and plasma AngII can not cross the intraocular space [24]. These findings suggest that the systemic RAS most likely influences the intraocular RAS through the RPE.

However, how the RPE would accomplish AT1R-dependent signaling transduction is unknown. It has been proposed that activation of AT1R by AngII triggers a variety of complex intracellular signaling events, such as activation of phospholipase-D [25], induction of phospholipase-C-γ (PLC) phosphorylation or activation of Gq protein-linked to PLC/inositol-1,4,5-trisphosphate (IP3), this last causing transient release of Ca^2+^from the endoplasmic reticulum (ER) [26]. The concept of store-operated transmembrane Ca^2+^entry during AngII stimulation in juxtaglomerular cells is well established [27], [28]. Other studies suggest the participation of voltage-activated calcium channels [29] or store-independent calcium-activated Ca^2+^channels [30] in mediating the influx of Ca^2+^by AngII. However, the identity of molecules responsible of such Ca^2+^influx remains controversial. In the RPE, different Ca^2+^channel types are functionally expressed potentially enabling the AngII-mediated Ca^2+^entry such as Ca_V_1.3, Orai, (transient-receptor-potential channel) TRPC1/4 and TRPV2 [31]–[33]. Among them, only TRPV2 channels were reported to be linked to a G-protein-coupled receptor and PLC coupled pathway [34]. For this reason, TRPV2 channels are attractive candidates to mediate AngII-elicited Ca^2+^responses.

In contrast to the pathology role of the RAS in the retina, which is well characterized, its physiological function is less understood [8]. The present study aimed to clarify the molecular mechanism by which systemic AngII modulates the RAS in the eye. Here, we present evidence that the transient-receptor-potential channel-V2 (TRPV2) and Atrap constitute important factors in the complex AngII-mediated signaling pathway in the RPE.

## Methods

### Animals and Ethical Approval

Atrap knockout (*Atrap* ^−/−^) adult mice (C57BL/6x129SvEv) from a local colony were used in the study. (Oppermann et al., 2010). *Atrap* ^−/−^ and *Atrap* ^+/+^littermates were used from heterozygous breeding pairs. All experimental procedures were performed following the guidelines approved by the Institutional Animal Care and Use Committee at University of Regensburg and the Association for Research in Vision and Ophthalmology (ARVO) statement for the use of animals in vision research. All animal experiments were formally approved by the German authorities (Regierung Oberpfalz) under the number 54-2532.1-06/10.

### Reverse Transcriptase-PCR (RT-PCR) Analysis

Expression of angiotensin receptor isoforms, Atrap and TRPV2 in cultured (mouse or porcine) RPE, retina, kidney and lung was carried out by isolation of total RNA either from cultured cells or from the corresponding tissues using the NucleoSpin RNAII kit (Macherey-Nagel, Düren, Germany). Reverse transcription was assessed using the RevertAid M-MuLV reverse transcriptase (Fermentas, St.Leon-Rot, Germany). Table 1 shows the oligonucleotide primers designed for detection of mRNA by PCR (gene, accession number, species oligonucleotide sequence 5′-3′ and size of PCR product). PCRs were performed at 94°C for 2 min, for 35 cycles at 94°C for 30 s, and at annealing temperatures of 57°C for 30 s and 72°C for 30 s. PCR products were visualized by loading onto agarose gels.

**Table 1:** Oligonucleotide primer sequences for RT-PCR and real -time quantitative RT-PCR.

Gene	Acc.No.	Species	Oligonucleotide sequence (5′-3′)	Size (bp)
Atrap	NM_009642	mouse	sense; CAGCTTGGCCCTTGTTCTCCAG antisense; CATCCTCAGCTTGCTGCTGAAG	194
At2r1a	NM_177322	mouse	sense; GCTGCTCTCCCGGACTTAA antisense; CGGAGGCGAGACTTCATTG	565
At2r1b	NM_175086	mouse	sense; GGGAGTAACAGAGACCAGACAAG antisense; CGGAGGCGAGACTTCATTG	565
Actb	NM_007393	mouse	sense; GTGGGGCGCCCCAGGCACCA antisense; CTCTTTGATGTCACGCACGATT	540
Atrap	XM_003127573	porcine	sense; GTCGACGCCATAAGTATGTTT antisense; CGGTACATGTGGTAGACGAG	182
At2r1	ENSSSCG00000011695	porcine	sense; TATTTGGGAACAGTTTGGTG antisense; GAAACTGACACTGGCTGAAG	203
GAPDH	NM_007393	porcine	sense; GACAACTTCGGCATCGTG antisense; GCTCAGTGTAGCCCAGGAT	340
TRPV2	XM_003132023	porcine	sense; ATCATCGCCTTTCACTGCC antisense; CAAAGTAGCTGTCCACGAAG	343

### Immunohistochemistry

After killing the mice, one eye from each subject was dissected and fixed in 4% (wt/vol) paraformaldehyde for 2 h at room temperature. Immunolabeling was performed on 3-µm paraffin sections, dewaxed, and treated in citrate buffer (citric acid trisodium salt: 1 mM; citric acid monohydrate: 0.2 mM) pH 6.0. Sections were incubated for 5 min in phosphate-buffered saline (PBS) containing Tween 0.2% pH 7.2 (PBS-Tween). Sections were permeabilized with blocking/permeabilization solution [10% (vol/vol), normal goat serum and 0.5% (vol/vol) Triton X-100 in 1×PBS] for 20 min. After three washing steps with PBS, eye sections were then labeled overnight at 4°C with anti-angiotensin II type 1 receptor (AT1R) antibody (ab18801; Abcam, Cambridge, UK) diluted 1∶100 or with anti-Atrap antibody [19], [20], diluted 1∶200 in 2% normal goat serum and 0.1% Triton X-100 in 1×PBS, pH 7.4. After three additional washing steps, sections were incubated for 1.5 h with secondary antibody conjugated with Alexa 488. (Invitrogen, Karlsruhe, Germany). Sections were mounted in confocal matrix (Micro Tech Lab, Graz, Austria) and examined on a Zeiss Axiovert 135 M microscope attached to a LSM 410 confocal laser scanning system (Carl Zeiss, Göttingen, Germany).

### Western Blotting

Western Blot analysis was performed as previously described in detail [35]. Cells were washed with PBS at 4°C three times followed by a centrifugation step of 5 min at 1000 g. The pellet was suspended in PBS-SDS buffer containing β mercaptoethanol (1∶100), a cocktail of protease inhibitors, tris-HCl pH 6.8, and glycerol [35], followed by ultrasound treatment twice for 10 seconds. Proteins were then heated at 90°C for 5 min and subjected to SDS-PAGE (10% gel). Proteins were blotted to a polyvinylidene difluoride membrane (GE Healthcare, Amersham Hybond-P). The blots were blocked in 5% nonfat dried milk in PBS/Tween 20 (0.05%) buffer for 1 h at room temperature. Primary antibodies were diluted as follows: anti-AT1R (1∶500), anti-Atrap (1∶500), anti-TRPV2 (1∶50) anti-β-actin (1∶5000). After overnight incubation at 4°C with primary antibodies, proteins were visualized using a horseradish peroxide-conjugated goat anti-rabbit- or anti-mouse- IgG secondary antibody (1∶5000; Cell Signaling Technology) and a Pierce ECL Western blotting substrate detection kit (Thermo Scientific). The images were digitized using an image analyzer (Alpha Innotech, FluorChemFC2). Each western blot experiment was repeated at least three times with different protein extracts. Densitometry was performed using the densitometry tool of the AlphaEase (FluorChemFC2) software which is implemented in the image analyzer software kit. Densitometric analysis was performed within the same blot using background substraction.

### Primary Culture of RPE Cells

Culture of mouse primary RPE cells (passage 0) was performed as described previously by [36]. Briefly, eyes from mice (2–3 months old) were enucleated, opened by a cut along the ora serrata and incubated in PBS-Ca^2+^/Mg^2+^free (Buffer I) for 20 min at 37°C. Eyes were transferred to buffer II (buffer I, 1 mM EDTA) for 25 min at 37°C. The retinae were carefully removed from the RPE in buffer I and the eye cups were transferred into the activated enzyme solution [buffer II, 1 U/ml papain (Sigma- 121 Aldrich), 3 mM L-cystein (Sigma-Aldrich) and 1 mg/ml 122 BSA (Sigma-Aldrich)]. After incubation for 23 min at 37°C, RPE cells were isolated and collected in culture medium (Hams/F-12/DMEM (PAA)): 10% fetal calf serum (PAA), 1% penicillin/streptomycin (PAA), 0.5 µl/ml insulin transferring selenium A (GIBCO), 1% nonessential amino acids (PAA) and 5 mM Hepes solution (PAA)] and maintained at 37°C and 5% CO_2_. Within 7 to10 days, cells from wild-type and *Atrap* ^−/−^ mice were all densely pigmented, showed uniform cubical morphology and were ready to use.

Culture of porcine primary RPE cells (passage 0) was performed as described previously by [7]. The eyes were opened by an incision along the ora serrata. The anterior parts of the eye including the retina were removed. Sheets of RPE cells were harvested in DMEM medium containing L-glutamine, 4.5 g/l glucose, and 110 mg/l sodium pyruvate, supplemented with 20% FCS, 100,000 U/l penicillin, and 100 mg/l streptomycin. RPE cells were suspended into a single suspension and plated out onto glass cover-slips. After 1–2 weeks, the cells formed a confluent monolayer of epithelial cells.

During the recording, unless otherwise specified, cells were bathed in Ringer’s solution composed of the following (in mM): 145 NaCl, 0.4 KH_2_PO_4_, 1.6 K_2_PO_4_, 5 glucose, 1 MgCl_2_, and 1.3 Ca^2+^-gluconate pH 7.4 (310–320 mOsm). AngII, U73122∶1-[6-[[(17β)-3-Methoxyestra-1,3,5(10)-trien-17-yl]amino]hexyl]-1*H*-pyrrole-2,5-dione, U73343∶1-[6-[[(17*β*)-3-Methoxyestra-1,3,5(10)-trien-17-yl]amino]hexyl]-2,5-pyrrolidinedione, xestospongin C and losartan, were purchased from Sigma. SKF96365∶1-[β-(3-(4-Methoxyphenyl) propoxy)-4-methoxyphenethyl]-1H-imidazole hydrochloride, 1-[2-(4-Methoxyphenyl)-2-[3-(4-methoxyphenyl)propoxy]ethyl]imidazole and cannabidiol were purchased from Tocris Bioscience.

### Small Interfering RNA

A mixture of three different small interfering RNAs (siRNA) were designed in duplexes of 25 nucleotides and synthesized by Invitrogen (Invitrogen, Paisley, UK). All three sense strands of the siRNA used to silence the porcine TRPV2 gene contained Alexa488 fluorescent dye. The three sense oligonucleotides are: 5′-Alexa 488-CACUGAGCUGGUGACCCUGAUGUAU-3′; 5′-Alexa 488-AGGUUCCUCAUCAACUUCCUGUGUU-3; 5′-Alexa 488-ACCUCCUUCGUGGACAGCUACUUUG-3′. A scrambled sequence siRNA double-stranded oligomer not homologous to any known gene (fluorescent Alexa 488-coupled oligonucleotide) served as a control. Transfection of primary porcine RPE cells was carried out 1 week after seeding using a lipophilic transfection reagent (Lipofectamine 2000, Invitrogen) in Opti-MEM. After 24–48 h, cells were used for patch clamping, Ca^2+^imaging or protein isolation.

### Measurements of Intracellular Free Ca^2+^

Measurements of intracellular free Ca^2+^were performed as we previously described [7]. TRPV2-RNAi or mock transfected cells were loaded with 5 µM fura-2 AM and transferred to a shallow recording chamber on stage of an inverted fluorescence microscope, Zeiss Axiovert 40 CFL (Carl Zeiss Göttingen, Germany) in conjunction with a Visitron Polychromator-System (Puchheim, Germany), equipped with Ca^2+^imaging system and continuously superfused with Ringeŕs solution. For Ca^2+^measurements, fluorescence was elicited at 340 nm and 380 nm excitation wavelengths, emission was filtered with a 510 nm filter and detected by a cooled charged-coupled device camera (CoolSnap, Visitron Systems, Puchheim, Germany). Data were collected and analyzed with MetaFlour software (Visitron Systems, Puchheim, Germany). We identified transfected cells by the expression of the fluorescent Alexa 488-coupled oligonucleotide and the given image was combined with the corresponding fura-2 AM image using ImageJ 1.44p software (National Institutes of Healths, USA) for data analysis. Cells were stimulated during 80 seconds by bath perfusion of 100 nM AngII or with different blockers. The ratio of F340/F380 was converted to approximate [Ca^2+^]_i_ as described by [37] using a fura-2 calibration. All experiments were conducted at room temperature.

### Statistical Analysis

Statistical significance for all calcium experiments was tested using ANOVA with Tukey’s multiple comparison post test. Statistical analysis using paired Student’s *t* tests was applied to determine whether densitometry measurements of western blot following a given treatment (mock or RNAi treatment) were significant. Data presented in bar graph shows mean ± SEM, p<0.05 was accepted as significant. n = number of cells from 4–9 (for poricine) or 9–16 (for mouse), independent experiments = cell cultures). The n-number were handled as individual cells because there was no indication that AngII-evoked Ca^2+^-raises spread among the cells due to gap-junction coupling. Only individual cells in a mono layer have responded to the AngII applications.

## Results

### AngII-evoked Ca^2+^Signaling in the RPE

**Figure 1:**
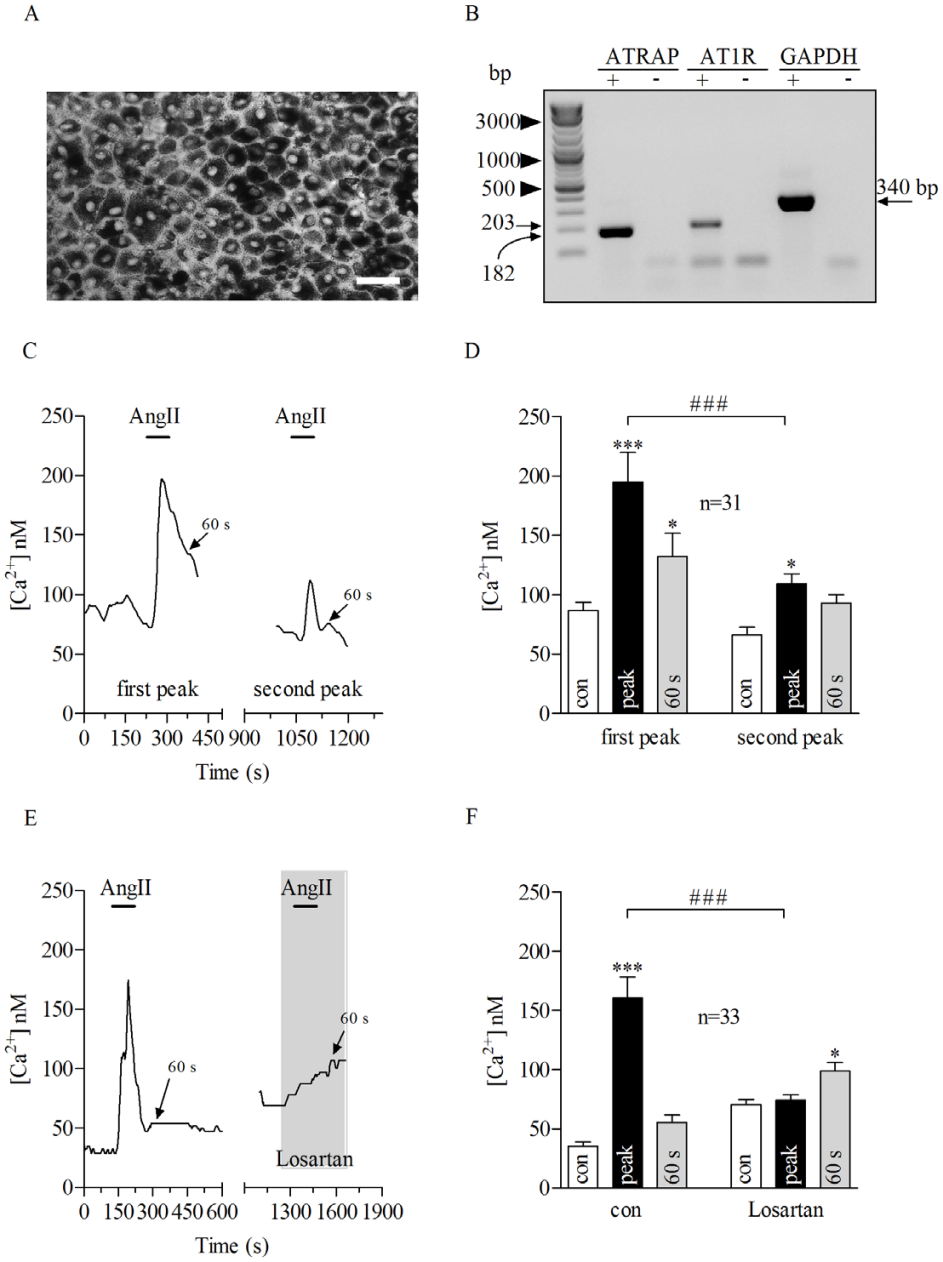
Porcine RPE cells constitute a suitable model to study AngII-evoked Ca^2+^ **response.** *A*: Freshly isolated porcine RPE (pRPE) in culture formed a pigmented monolayer. Scale bar, 40 µm. *B*: RT-PCR using mRNA from cultured pRPE cells expressed both Atrap (182 bp) and AT1R (203 bp), GAPDH (340 bp) was used as control. *C*: Bath application of AngII at 100 nM for 80 seconds (bars) evoked Ca^2+^responses in a pRPE cell. In the same cell, after washing out 100 nM AngII (until [Ca^2+^]_I_ returned back to resting levels) further application of AngII led to a second rise in [Ca^2+^]_i_. *D*: Summary of data from experiments shown in *C*. *E*: Losartan at 10 µM inhibited AngII-evoked Ca^2+^responses in pRPE cells. On the left panel it is shown the effect of AngII application under control conditions. In the same cell, after washing out AngII (until [Ca^2+^]_I_ returned back to resting levels) AngII was applied in the presence of losartan (right panel). The wash out is not shown and the x-axis is interrupted accordingly between the panels. Note that losartan itself led to an increase in intracellular free Ca^2+^. *F*: Summary of data from experiments shown in *E*. Bars in Fig. 1D and F represent means ± SEM for AngII-evoked Ca^2+^responses before (open bars) during the peak (black bars) and at 60 s after the maximum AngII-elicited calcium response (gray bars). * *p*<0.05, (***; ###) *p*<0.0001; repeated measures ANOVA. n = number of cells from 4 (*D*) or 7 (*F*) independent experiments.

In a recent study we showed that cultured porcine RPE (pRPE) represent a reliable model to study AngII signaling in the RPE [7]. In particular, as it is shown in Fig. 1A, freshly isolated pRPE cells form a tight and pigmented monolayer resembling the native architecture of the retinal epithelium. In addition, these cells showed a robust expression of AT1R and Atrap as demonstrated by RT-PCR (Fig. 1B). Application of AngII (100 nM) led to an increase in intracellular free Ca^2+^(Fig. 1C and 1D) which lasted longer than the application period of AngII resulting in a delayed recovery phase. Quantifications of the intracellular Ca^2+^concentration were performed at the resting (before AngII application), peak (during AngII) and 60 s after the Ca^2+^-peak (delayed recovery phase) for all the experiments. In all control experiments, as they will be shown later on (non-transfected and transfected porcine or non-transfected mouse RPE cells) at 60 s after the Ca^2+^-peak, the intracellular calcium differed to the resting Ca^2+^by 23–50 nM (p>0.05). In order to have an internal control for each cell in experiments using different blockers, we used an experimental paradigm consisting of a double application of AngII in sequence. Control experiments were performed to show that repeated AngII stimulation for 80 seconds leads to comparable Ca^2+^transients. Application of AngII at 100 nM to pRPE cells with 7 minutes of wash out between applications (until [Ca^2+^]_i_ returned back to the resting level) led to transient rise in [Ca^2+^]_i_ concentration (Fig. 1C and D). Importantly, the AngII-evoked calcium mobilization in pRPE was due to specific activation of AT1 receptor by AngII, since bath application of the AT1 receptor blocker losartan at 10 µM abolished the AngII-induced calcium increase in pRPE (Fig. 1E and F).

**Figure 2:**
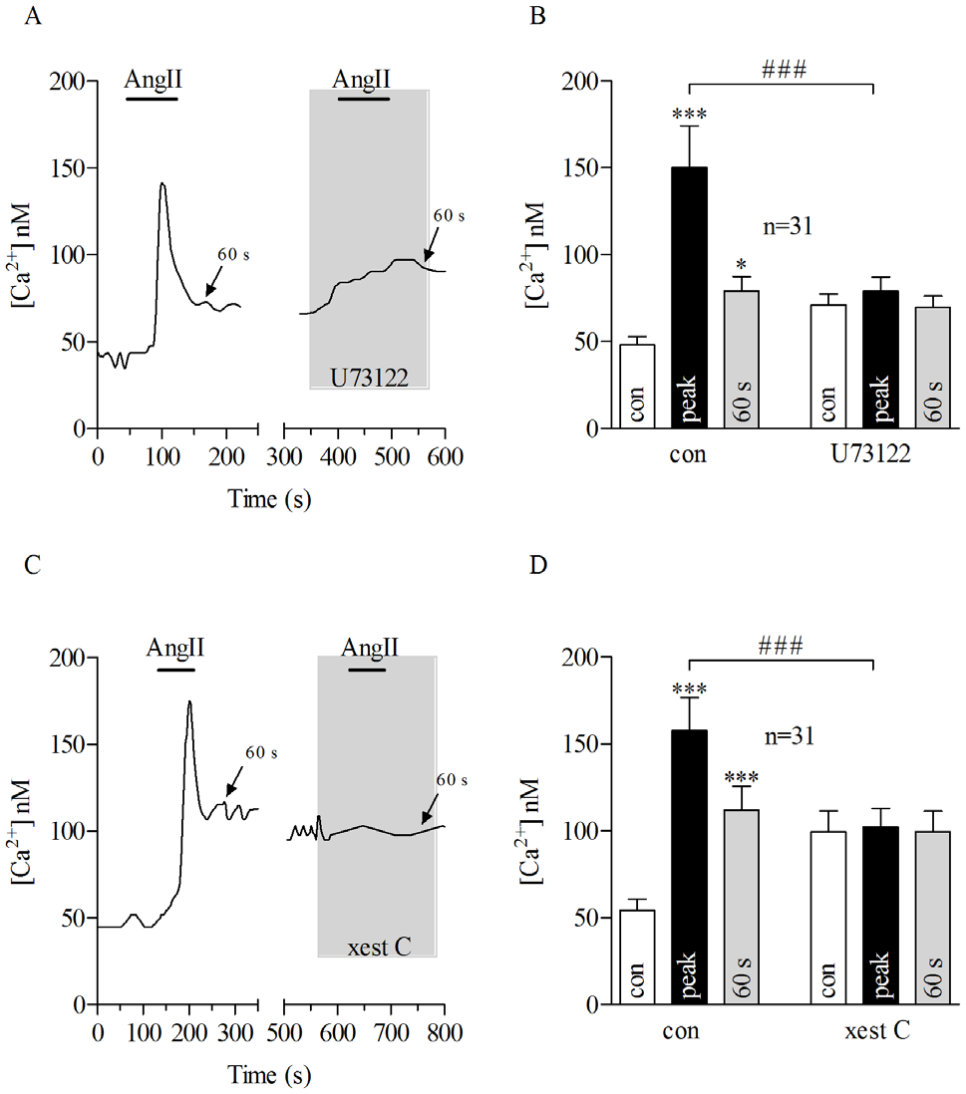
AngII-evoked Ca^2+^ response is mediated by PLC/IP3 pathway in porcine RPE cells. Application of AngII for 80 seconds (bars) caused transient Ca^2+^response in pRPE cells. *A:* Bath application of 100 nM AngII (bars) together with 10 µM U73122 (gray shadow), a phospholipase C (PLC) blocker abolished AngII-evoked Ca^2+^signal in pRPE cells. On the left panels of Figures A and C, it is shown the effect of AngII application under control conditions. In the same cell, after washing out first AngII application (until [Ca^2+^]_I_ returned back to basal levels) it was further perfused 100 nM of AngII in the presence of U73122 (right panel in A) or xest C (right panel in C). The wash out is not shown and the x-axis is interrupted accordingly between the panels. Note that application of U73122 alone led to a slight increase in intracellular free Ca^2+^. *B*: summary of data from experiments shown in *A*. *C*: Co-application of 100 nM AngII (bar) with the IP3 blocker xestospongin C (xest C) (gray shadow) at 10 µM reduced AngII-evoked Ca^2+^response in pRPE cells. *D*: summary of data from experiments shown in *C*. Bars in Fig. 2B and D represent means ± SEM for AngII-evoked Ca^2+^responses before (open bars) during the peak (black bars) and at 60 s after the maximum AngII-elicited calcium response (gray bars). *p<0.05, (***; ###) *p*<0.0001; repeated measures ANOVA. n = number of cells from 4 (*B*) or 5 (*D*) independent experiments.

The transient calcium response triggered by AngII stimulation might be the result of Ca^2+^release from the endoplasmic reticulum (ER), and the subsequent sustained Ca^2+^entry from the extracellular compartment or a combination of both [38], [39]. The contribution of each of these pathways was tested in the porcine RPE model. In these experiments AngII was applied first alone and after wash out until [Ca^2+^]_i_ has returned back to the resting level, then AngII was applied a second time in the presence of a blocker for these two pathways. Perfusion of the phospholipase C (PLC) blocker U73122 (10 µM) completely inhibited the AngII-dependent calcium increase observed in pRPE (Fig. 2A and B). Unlike U73122, application of the inactive analog U73343 at 10 µM did not affect the AngII-evoked calcium response (Fig. S1). In addition, application of 1 µM of xestospongin C, a selective and reversible blocker of inositol-1,4,5-trisphosphate (IP3) receptor, reduced the AngII-induced Ca^2+^response in pRPE cells (Fig. 2 C and D).

### Involvement of Angiotensin-receptor-associated Protein (Atrap)

**Figure 3:**
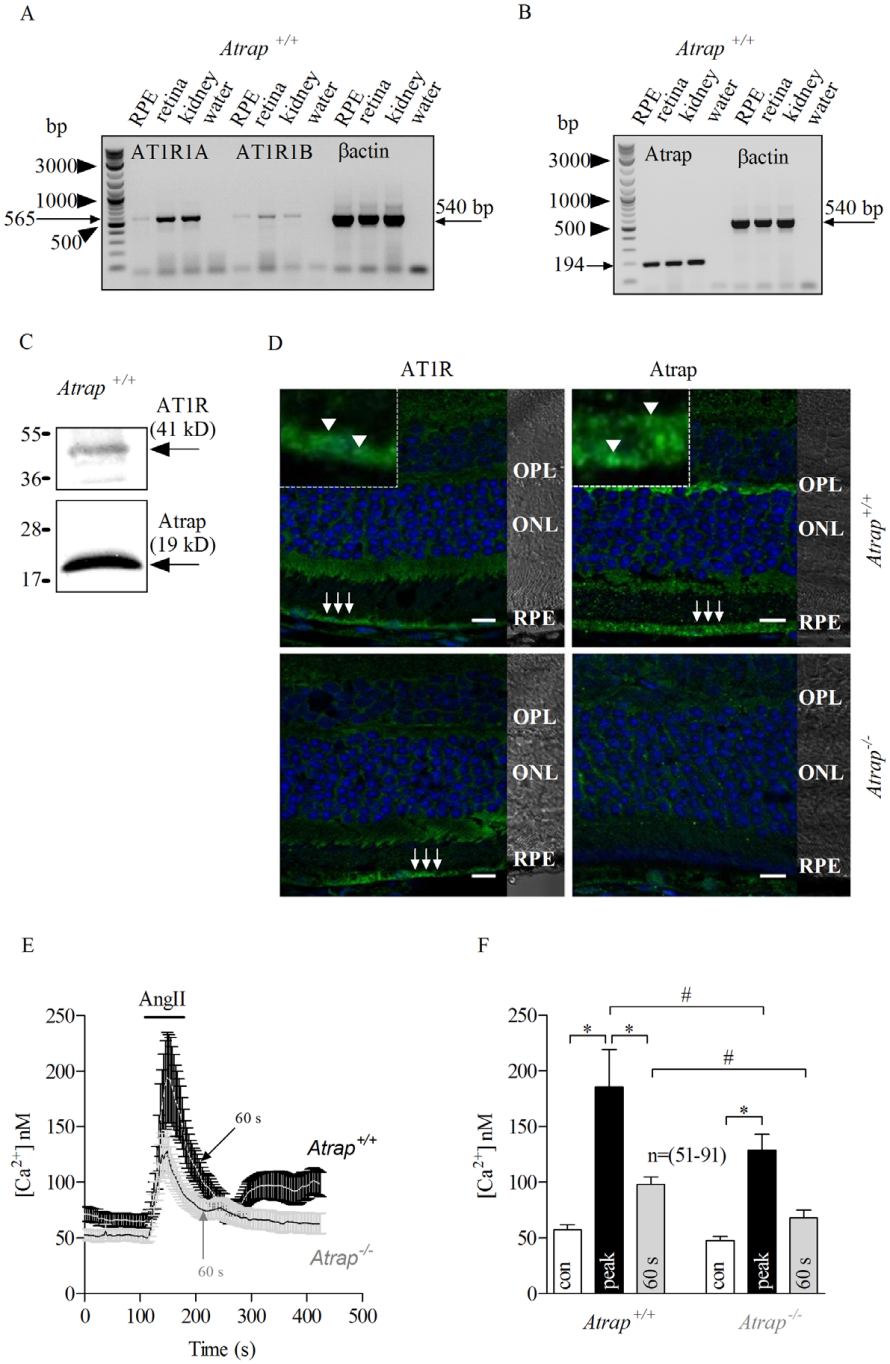
Lack of Atrap reduces AngII-mediated Ca^2+^ signaling in mouse RPE cells. RT-PCR from freshly isolated mouse *Atrap^+/+^*RPE cells shows expression of both AT1 receptor paralogs (AT1R 1A and AT1R 1B; 565 bp) (*A*) and Atrap; 194 bp (*B*). βactin mRNA (540 bp) from retinal and kidney tissues served as control. *C*: Western Blot analysis indicates the expression of both AT1R and Atrap in mouse RPE cells. *D*: Immunostaining of *Atrap* ^+/+^and *Atrap* ^−/−^ mouse retinas using antibodies against AT1R (arrows, left panels) and Atrap (arrows, right panels). Insets represent an enlarged confocal area around the arrows, indicating basolateral localization of AT1R (arrowheads, upper left panel) and Atrap (arrowheads, upper right panel) at both the basolateral and the apical side. Differential Interference Contrast (DIC) image illustrates the retinal layers as outer plexiform layer (OPL), outer nuclear layer (ONL) and retinal pigment epithelium (RPE). *E*: Traces show transient Ca^2+^response from *Atrap* ^+/+^(mean, white line; SEM in black) and *Atrap* ^−/−^ (mean, black line; SEM in gray) RPE cells upon 80 seconds AngII (100 nM) stimulation (bars). AngII-evoked Ca^2+^response was smaller in *Atrap* ^−/−^ mouse cells than that from *Atrap* ^+/+^. *F*: Summary of data from experiments shown in *E*. Bars in Fig. 3F represent means ± SEM for AngII-evoked responses before (open bars) during the peak (black bars) and at 60 s after the maximum AngII-elicited calcium response (gray bars). (*; #) *p*<0.05; ANOVA analysis. n = number of cells from 15 independent experiments.

Atrap protein interacts directly with angiotensin-II type 1 receptor (AT1R) [40], playing a role in the underlying AngII-stimulated signal transduction cascades. Due to the lack of both pharmacological blockers for porcine Atrap and a gene sequence database to design siRNA capable of silencing porcine Atrap, we used a knock-out mouse model for Atrap to study the role of this protein in the AngII-stimulated Ca^2+^-signals in the RPE. We detected the expression of the two AT1 receptor paralogs (AT1R 1A and AT1R 1B) by RT-PCR in isolated mouse (*Atrap* ^+/+^and *Atrap* ^−/−^) RPE cells (Fig. 3A) and Atrap protein (Fig. 3B) in isolated mouse wild type RPE cells. By means of RT-PCR in isolated mouse (*Atrap* ^+/+^) RPE cells, we detected the expression of the two AT1 receptor paralogs (AT1R 1A and AT1R 1B) (Fig. 3A) and also a robust expression of Atrap (Fig. 3B). Moreover, AT1R and Atrap proteins were detected by western blotting as shown in (Fig. 3C). AT1R expression was also demonstrated by immunohistochemistry in RPE cells from both wild type (*Atrap* ^+/+^) and Atrap-deficient (*Atrap* ^−/−^) mice (Fig. 3D, left panels) while Atrap protein expression was detected in the outer plexiform layer and in the RPE from *Atrap* ^+/+^mice (Fig. 3D, right panels). Notably, AT1R and Atrap were both expressed at or near the basolateral membrane of the RPE (Fig. 1D insets) in the wild-type mouse. Atrap staining was also visible at the apical membrane of the RPE. In order to study the AngII-evoked calcium transients, we cultured RPE cells isolated from *Atrap* ^+/+^and *Atrap* ^−/−^ mice and measured the AngII-evoked calcium mobilization using the Ca^2+^indicator fura-2 AM. Bath application of 100 nM AngII increased the intracellular calcium concentration [Ca^2+^]_i_ with an initial peak followed by a delayed recovery phase in *Atrap* ^+/+^RPE cells, whereas in *Atrap* ^−/−^ RPE cells, the peak was smaller with the sustained component concomitantly smaller (Fig. 3E and F). Remarkably, the wild-type mouse RPE cells showed a different behavior of the AngII-evoked Ca^2+^response compared to that in the porcine cells. In the mouse cells a second rise of intracellular free Ca^2+^occurred two minutes after AngII application. Since it is likely that it is a species difference we did not further investigate this effect. When comparing the AngII responses in the mouse RPE cells, we found that Ca^2+^levels rose from a resting concentration of 57.13±5.55 nM to a peak of 185.46±34.03 nM in wild-type cells, in contrast to a resting Ca^2+^concentration of 47.72±3.55 nM to a peak of 128.94±14.03 nM in *Atrap^−/−^* mouse cells. The increase in concentration was significantly greater in wild-type cells (Fig. 3E).

### Functional Expression of TRPV2 Channels in the Porcine RPE Cells

**Figure 4:**
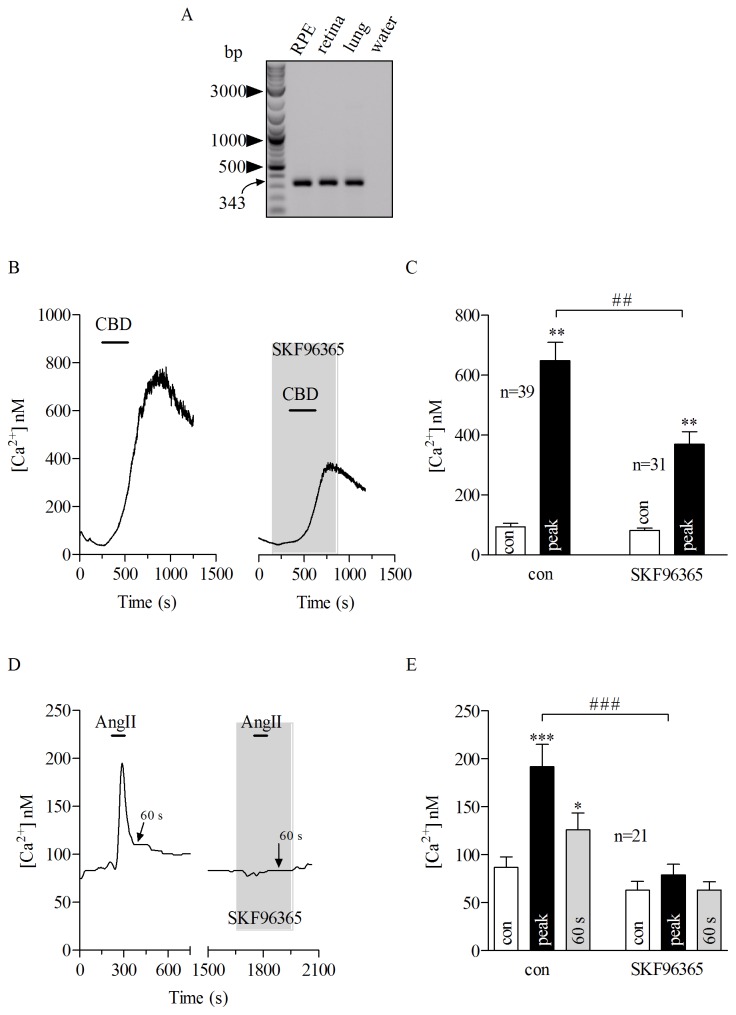
TRPV2 channels is present in porcine RPE cells. *A:* RT-PCR using mRNA from cultured porcine (pRPE) cells. mRNA from retinal and lung tissues were used as control. Porcine RPE robustly expressed TRPV2 (343 bp). *B*: *left panel,* 15 µM cannabidiol (CBD) was applied for a period of 10 min (bars) where it caused a reversible Ca^2+^response in a pRPE cell. *Right panel:* Bath application of 15 µM CBD (bar) together with 100 µM SKF96365, a TRPV channel inhibitor (gray shadow) reduced the CBD-evoked Ca^2+^signal in another pRPE cell. *C*: summary of data from experiments shown in *B*. *D*: Application of AngII for 80 seconds (bars) caused transient Ca^2+^response in a pRPE cell. In the same cell, after washing out AngII until [Ca^2+^]_I_ returned back to resting levels, AngII-mediated Ca^2+^signal was prevented by application of 100 µM SKF96365 (gray shadow). Note the wash out is not shown and the x-axis is interrupted accordingly between the panels. *E*: summary of data from experiments shown in *D*. Bars in Fig. 4C and E represent respectively means ± SEM for CBD- or AngII-evoked Ca^2+^responses before (open bars) during the peak (black bars) and at 60 s after the maximum AngII-elicited calcium response (gray bars). (**; ##) *p*<0.001, (***; ###) *p*<0.0001; repeated measures ANOVA. n = number of cells from 5 (*C*) or 6 (*E*) independent experiments.

When compared to wild type (*Atrap^+/+^*), RPE cells from *Atrap^−/−^* mouse showed two main features in the AngII-evoked Ca^2+^signals: a reduced initial peak and sustained component remarkably smaller. Classical Ca^2+^signals elicited from G protein-coupled receptors comprises an initial peak that results from the release of Ca^2+^from the cytosolic stores, while the subsequently occurring phase results from the influx of extracellular Ca^2+^into the cell. In the next step we aimed to identify the molecular entity contributing to the delayed recovery phase which, most likely, results from activation of Ca^2+^-conducting ion channels. Human RPE cells express functional IGF-1 receptor-activated TRPV2 channels [33] which are also known to be activated by G protein-coupled receptors. Along this line, TRPV2 channels appeared as possible candidates to contribute to the delayed recovery phase of AngII-evoked Ca^2+^rises in the RPE cells. With RT-PCR we found that porcine RPE cells also express TRPV2 channels (Fig. 4A). In order to show the functional expression of TRPV2 channel in pRPE cells, we analyzed the Ca^2+^response to cannabidiol (a TRPV channel activator) and SKF96365 (a TRPV channel inhibitor). This was done in two separated set of experiments. In one cannabidiol was applied alone and in the second set cannabidiol was applied in the presence of SKF96365. Application of 15 µM cannabidiol, a broad range TRPV channel opener [41], rose the intracellular free Ca^2+^from a resting concentration of 93.6±11.9 nM to a peak of 648.1±61.8 nM (Fig. 4B and C, left). Importantly, pre-incubating the cells with the TRPV2 inhibitor SKF96365 at100 µM, reduced the cannabidiol-evoked Ca^2+^rise by 48.61% (from a resting concentration of 81.6±7.7 nM to a peak of 369.1±42.9 nM ) when compared to control (Fig. 4B and C). Next we tested the effect of SKF96365 on AngII-induced Ca^2+^transients. In these experiments AngII was applied first alone and after wash out until [Ca^2+^]_i_ has returned back to the resting level, then AngII was applied a second time in the presence of SKF96365. SKF96365 at 100 µM abolished the AngII-evoked Ca^2+^rise (Fig. 4 D and E), again suggesting that TRPV2 plays a role in the AngII-evoked Ca^2+^signaling in pRPE cells (delayed recovery phase control: 125.9±17.9 nM and delayed recovery phase with SKF96365∶63.2±8.5 nM). In summary, this pharmacologic profile suggests that porcine RPE cells express functional SKF96365-sensitive TRPV2 channels likely contributing to the delay in recovery phase of AngII-induced Ca^2+^rises.

### Contribution of TRPV2 to Ca^2+^Entry Upon AngII Stimulation

Although the pharmacology approach we used here strongly suggests that TRP channels are responsible for generating the sustained Ca^2+^rise upon AngII stimulation, concerns about the low specificity of the blocker may still exit. In order to prove this more conclusively, we used RNAi approach against TRPV2.

**Figure 5:**
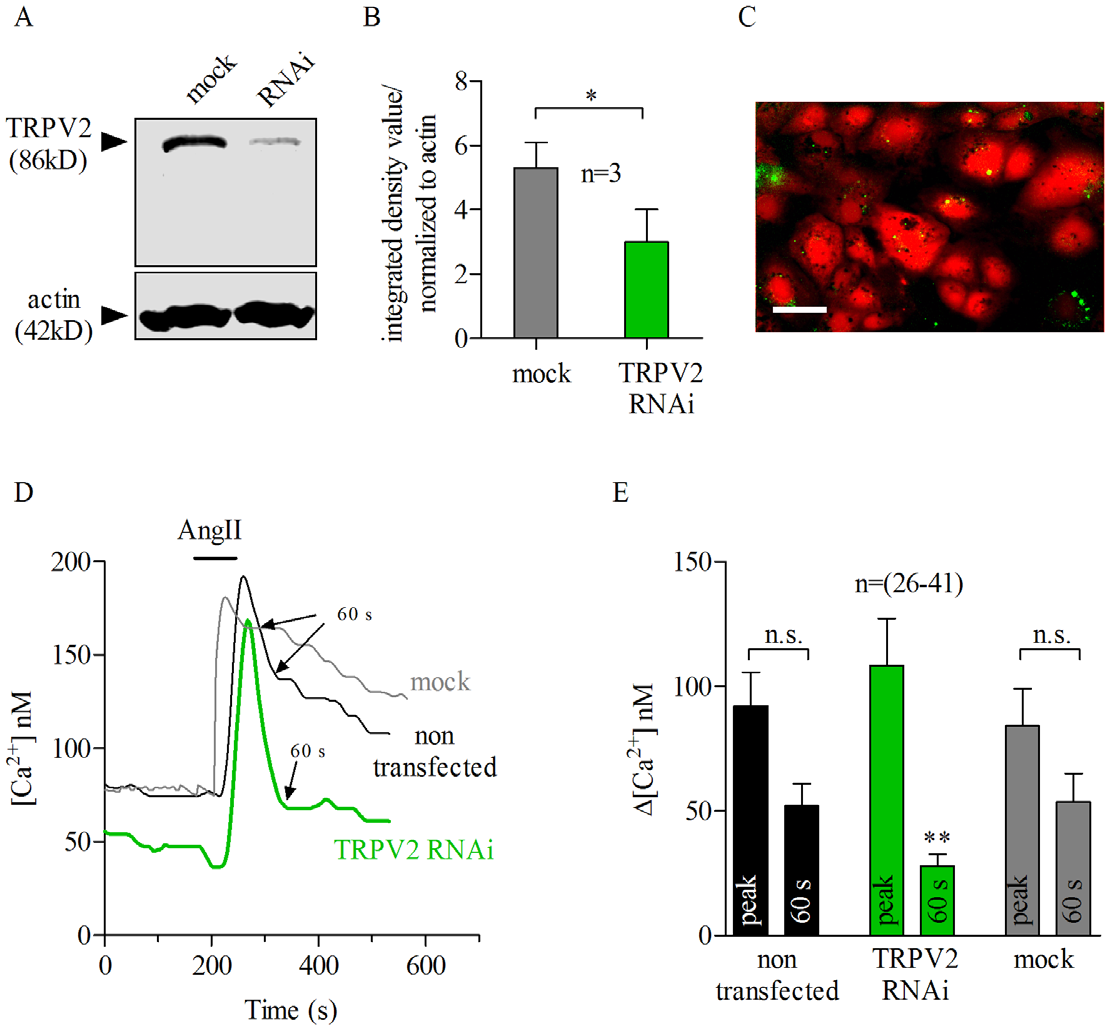
AngII-evoked Ca^2+^ response is mediated by TRPV2-dependent Ca^2+^ influx in porcine RPE cells. *A*: Western blot analysis indicated successful suppression of TRPV2 channel by RNAi. Transfection with mock RNA served as control. *B*: Densitometry analysis of each lane in the western blot shown in *A*. TRPV2- RNAi treated cells (green) showed a down-regulation of TRPV2 protein in about 55% compared to mock transfected cells (gray). TRPV2 bands in each group (mock and RNAi) were normalized to actin. Bars represent means ± SEM; * *p*<0.05, paired *t* test. Densitometry analysis results from 3 different blots; n = 3. *C*: Representative Fura 2-AM loaded cells (in red) and cells transfected with either TRPV2-RNAi or mock RNA- bound to Alexa 488 fluorescent dye (green dots) (see methods). Scale bar, 30 µm. *D*: TRPV2-RNAi treated cells (green trace) showed a quick reduction (within 60 s, arrow) of AngII-evoked Ca^2+^(bar) elevation when compared to non-transfected (black trace) or mock-transfected cells (gray trace). Application of AngII at 100 nM for 80 seconds (bar) evokes similar Ca^2+^responses in mock (gray trace) or non-transfected (black trace) porcine RPE cells. Transfection with mock RNA (gray trace) did not alter the sustained AngII-evoked Ca^2+^elevation (delayed recovery phase) as TRPV2-RNAi did. Sixty seconds after the maximum AngII-elicited calcium response is shown by arrows. *E*: Summary of data from experiments shown in *D*. Black (for non-transfected cells), green (for TRPV2-RNAi) and gray (for mock) transfected cells. Bars represent means ± SEM of the difference between AngII-evoked Ca^2+^signal at the peak and 60 seconds after the peak respectively. (**) *p*<0.001; repeated measures ANOVA. n.s = not significant. n = number of cells from 26–41 (*E*) independent experiments. *A:* Application of AngII (100 nM) for 80 seconds (bars) caused transient Ca^2+^response in pRPE cells. Bath application of 100 nM AngII (bars) produced a Ca^2+^response that was not abolished by co-application of 10 µM U73343 (gray shadow), the inactive analog of the phospholipase C (PLC) blocker U73122. *B*: summary of data from experiments shown in *A.* Bars in Fig. 2B represent means ± SEM for AngII-evoked Ca^2+^responses before (open bars) during the peak (black bars) and at 60 s after the maximum AngII-elicited calcium response (gray bars). (*; #) *p*<0.05, ** *p*<0.0001; repeated measures ANOVA. n = number of cells from 9 independent experiments.

The RNAi specifically targeting TRPV2 (TRPV2-RNAi) reduced endogenous TRPV2 channel protein levels to about 55% (green bar) of those in mock transfected cells (gray bar), according to densitometry analysis (Fig. 5B) of western blots (Fig. 5A). We measured the intracellular calcium response elicited by AngII in pRPE cells transfected with fluorescence-tagged TRPV2-RNAi and compared with non-transfected or fluorescence-tagged mock transfected cells using the Ca^2+^indicator fura-2 AM (Fig. 5C: green dots are fluorescence-tagged TRPV2-RNAi cells loaded with fura-2 AM in red). Using this technique, AngII-evoked Ca^2+^rises in TRPV2-RNAi loaded cells could be compared with non-transfected cells at the same time. The same was done in control experiments with mock transfection. Thus the effect of TRPV2 knock-down was simultaneously analyzed against two controls: non-transfected and mock-transfected cells. The Ca^2+^peak response to 100 nM AngII was similar, in size and shape, in these three groups (Fig. 5D). However, the sustained Ca^2+^phase (delayed recovery phase ) in TRPV2-RNAi cells occurring 60 seconds after the peak AngII-response (arrows) was significantly reduced when compared with non-transfected or mock transfected cells (Fig. 5D and E, green). Sixty seconds after the maximum AngII-elicited calcium response (arrows), mock or non-transfected cells had comparable cytosolic Ca^2+^levels (delayed recovery phase), which were much higher than those observed for TRPV2-RNAi treated cells at 60 seconds (Fig. 5D and E). Together, these findings indicate that TRPV2 channel is involved in the AngII-evoked calcium response in porcine RPE cells.

## Discussion

The present study was performed to evaluate the calcium signaling pathway triggered by AngII in the RPE. The most remarkable result presented here is that AngII-dependent Ca^2+^responses in the RPE involves both calcium mobilization from intracellular stores and extracellular Ca^2+^entry, most likely through TRPV2 channel activation. Furthermore, a new role of Atrap for AngII-dependent Ca^2+^-signalling was found in the RPE.

Milenkovic et al. (2010) reported recently that systemic infusion of AngII into mice caused a significant decrease in renin expression in the kidney and a reduction of the renin mRNA levels in both RPE cells and neuronal retina, whereas systemic application of ACE inhibitor increased the renin expression in the RPE by 20-fold [7]. Since previous studies showed that AngII from the plasma cannot enter the eye [24], it was suggested that systemic AngII modulates local intraocular renin production through AT1R activation in the RPE [7]. In order to analyze the molecular mechanism contributing to the signaling cascade leading to AngII-mediated effects, we performed Ca^2+^-imaging experiments. As we have reported previously cultured pRPE cells showed biphasic increases in intracellular free Ca^2+^upon stimulation by AngII. Since in the presence of the specific AT1R blocker losartan AngII failed to evoke Ca^2+^transients, the AngII-stimulated effect was due to specific activation of AT1R receptors and not AT2 receptors, which is in accordance with previously published data [7].

Our study shows that AngII stimulation of AT1R receptor leads to release of Ca^2+^from the ER through the activation of PLC and concomitant generation of IP3. Incubation with the PLC blocker U73122 completely abolished the AngII Ca^2+^response and inhibition of calcium release by using the IP3R blocker xestospongine C also prevented the calcium rise evoked by AngII. In agreement with our finding, Fellner and Arendshorst (2005), using calcium imaging techniques on isolated afferent arterioles reported that the peak response to AngII was attenuated by inhibiting the IP3 receptors with 8-(*N*,*N*-diethylamino) octyl 3,4,5-trimethoxybenzoate (TMB). These IP3R blockers also inhibited AngII-mediated AT1R induced calcium release from the ER through PLC/IP3 pathways in preglomerular renal vascular smooth muscle cells [42]. In addition, there is functional evidence in support of Ca^2+^mobilization in vivo, since TMB decreased AngII-dependent renal vasoconstriction [43]. Praddaude et al. (2009), using calcium imaging on mouse RPE sheets, showed that AngII stimulation of AT1R also increases [Ca^2+^]_i_ in a biphasic manner, with both an initial peak and plateau phase. Thus in the RPE as in other AngII sensitive cells the AngII-dependent rise of [Ca^2+^]_i_ depends on release of Ca^2+^from cytosolic stores.

Since Atrap is a protein bound to the AT1R, it is possible that Atrap is involved in the ignition of the cascade of AT1R signaling in the RPE. We found that mouse RPE cells in situ and in vitro and porcine RPE in vitro express Atrap. Both AT1R and Atrap were found at the basolateral membrane of the RPE in the wild-type. This implies a functional association of the proteins. However, a proportion of Atrap was also found at the apical membrane of the RPE which implies possible functions of Atrap other than AngII-signaling. *Atrap^−/−^* mice demonstrated AT1R expression as it was shown by immunohistochemistry on retina sections. In addition to that cultured RPE cells from *Atrap^−/−^* mice show AngII-evoked Ca^2+^responses which prove that the functional expression of AT1R is maintained in the cell culture. Thus, we can use mouse RPE cells to study AngII-mediated Ca^2+^transients in the absence of Atrap. Also in the mouse RPE, AngII led to a biphasic increase in intracellular Ca^2+^, which consists of an initial peak followed by sustained phases. We found that *Atrap^−/−^* RPE cells showed a smaller Ca^2+^peak and a smaller intracellular Ca^2+^during the recovery phase (measured at 60 s), which implies that Atrap plays a role in mediating the different events of AngII-mediated Ca^2+^signaling. This observation points to a new function of Atrap. Atrap had been considered to be a negative regulator of the surface expression of AT1R [20]. This does not seem to be the case in RPE cells, at least as shown by the similar staining of AT1R in both wild-type and *Atrap^−/−^* retina sections. The smaller Ca^2+^signals would indicate more likely a role as a positive regulator of AT1R-mediated signaling cascade. Due to the limited material of the RPE cells in mice it was not possible to measure the AT1R surface expression in wild-type and Atrap knock-out mice. Because the absence of Atrap reduces the Ca^2+^peak, Atrap might directly influence the mechanism of Ca^2+^signaling, perhaps by depleting cytosolic Ca^2+^stores. However, without knowing other proteins with which Atrap might interact it is uncertain what the nature of this influence might be and in which parts of the signaling pathway Atrap might be involved in.

In general, less well understood are the mechanisms involved in the activation of Ca^2+^entry pathways following stimulation by AngII that generate a plateau phase, both in RPE cells and in other cell types. In renal vascular smooth muscle cells, removal of extracellular Ca^2+^attenuated the AngII-mediated peak response to about 50% of the control value, with the remaining proportion likely stemming from intracellular stores [42]. The discussion above implicates that the plateau phase of the AngII-evoked Ca^2+^transients likely results from a Ca^2+^influx into the cell through activation of Ca^2+^conducting ion channels. It has been long known that many TRP channels can be stimulated subsequent to PLC activation by G protein-coupled receptors, by accumulation of DAG in the membrane after PLC activation, by tyrosine kinase receptors, or by intracellular Ca^2+^directly, this last feature a property of the majority of these ion channels [44]. Freshly isolated human RPE cells and ARPE-19 cells express TRPV1, 2, 3 and 4 [33] and possibly TRPV5 and 6 [45]. Among those, TRPV2 was found to be functionally expressed. TRPV2 is regulated by heat, growth factors, and other ligands [33], [46]–[49]. Indeed, the study from Monet et al. (2009) using pertussis-toxin (a G_i_ and G_o_-protein blocker) in CHO and HEK cells suggested that lysophosphatydilcholine-(LPC) dependent TRPV2 activation is mediated by G-protein pathways. In the human RPE, functional expression of TRPV2 channels has also been shown [33]. Thus, it is likely that in the RPE a comparable mechanism couples the activation of AT1R with the generation of a delayed recovery phase through activation of TRPV2 channels. In order to test this hypothesis we first checked whether porcine RPE cells functionally express TRPV2 by pharmacological means. Application of cannabidiol strongly increases intracellular Ca^2+^in porcine RPE cells. Although cannabidiol is known to activate TRPV1,2,3 and 4, it is more selective to TRPV2 [41], [50]–[53]. Importantly, SKF96365, a blocker of TRPV2 channels, reduced cannabidiol-evoked Ca^2+^signaling. SKF96365 is known to block TRPC channels, T-type Ca^2+^channels and TRPV2 channels [54]–[58]. Among these Ca^2+^channels only TRPV2 is known to be activated by cannabidiol. Thus, this experiment clearly demonstrates the functional presence of TRPV2 channels in porcine RPE cells. However, the cannabidiol response was not fully blocked by SKF96365. This can be explained by the fact that the RPE also express the TRPV 1, 3 and 4 channels which are also known to be activated by cannabidiol. It is likely that the SKF96365-insensitive part of the cannabidiol response is due the activation of TRPV1, 3 or 4 channels.

Based on the pharmacologic analysis discussed above SKF96365 was used to gain insight to whether TRPV2 channels might contribute to the delayed recovery phase of AngII-evoked Ca^2+^rises. Indeed, SKF96365 prevented the AngII-evoked Ca^2+^response in pRPE which strongly reinforces the concept of a role for Ca^2+^entry from the extracellular compartments and implies a contribution of TRPV2 channels. However, nothing has been previously reported about TRPV2 channel activation by AngII. Because both mRNA and protein TRPV2 were shown to be expressed in pRPE cells, we investigated the implications of TRPV2 activation on the AngII-mediated Ca^2+^signaling in RPE cells by means of RNAi techniques. Our calcium imaging data clearly show that knockdown of endogenous TRPV2 channels with TRPV2-RNAi strongly reduced the delay in the recovery phase AngII-mediated Ca^2+^response (later 60 seconds after the peak burst) and showed the same magnitude as in SKF96365-treated cells. Thus, the remaining Ca^2+^elicited after AngII stimulation in RNAi treated cells very likely comes from Ca^2+^release from the stores. This observation strongly indicates that TRPV2 contributes to the AngII-induced Ca^2+^entry in the RPE.

The AngII signaling at the RPE is of importance to understand inflammatory and/or neovascular diseases of the retina. In recent years the effects of AngII signaling in diseases such as diabetic retinopathy or age-related macular degeneration has gained increasing attention [8], [10]–[17], [59]–[61]. The discovery of a local renin-angiotensin system in the retina has opened new ways of understanding patho-physiological processes leading to inflammation or neovascularisation [8]. The local AngII and AT1R signaling was found to participate in these processes. However, recent observations point also to the participation of AT2R and AngI reaction products such as Ang1-7 [8], [59]. The latter appeared to show anti-inflammatory and anti-angiogenic effects. Thus the exploration of the local renin-angiotensin system of the retina will open new ways in the development of therapies. However, in contrast to these promising observations the systemic application of drugs which modulate the renin-angiotensin system did not show the expected effects or even adverse effects [62]. One reason might be the AngII signaling at the RPE. The RPE is able to react upon changes in the AngII levels in the plasma via basolateral AT1R [7] and possible through the connected signaling via Atrap and TRPV2 channels. This AngII signaling pathway leads to a reduced renin production by the RPE and decreased renin activity in the retina [7]. Thus the AngII signaling at the RPE connects the local renin-angiotensin system with systemic renin-angiotensin system. This understanding is of utmost importance to develop therapies which target the local renin-angiotensin system of the retina.

In summary the following picture of AT1R signaling in RPE cells emerges from our data. Activation of AT1R leads to an increase in intracellular free Ca^2+^by activation of PLC and subsequent release of Ca^2+^from intracellular Ca^2+^stores via activation of IP3-receptors. This leads to an initial peak of the Ca^2+^response followed by a Ca^2+^influx into the cell through TRPV2 channels, which yields a sustained Ca^2+^increase. The generation of the initial peak and the sustained response requires the activation of Atrap. In particular, the sustained Ca^2+^response requires the presence of Atrap, as suggested by the fact that it was almost abolished in RPE cells from Atrap knock-out mice. This also appears to be the first physiological demonstration of a role of TRPV2 in the intraocular RAS. Together, our results constitute a significant and novel addition to our understanding regarding how the systemic RAS interacts with the RAS in the eye.

## Supporting Information

Figure S1 **Effect of U73343, the inactive analof of U73122, on AngII-evoked Ca^2+^response.** *A:* Application of AngII (100 nM) for 80 seconds (bars) caused transient Ca^2+^response in pRPE cells. Bath application of 100 nM AngII (bars) produced a Ca^2+^response that was not abolished by co-application of 10 µM U73343 (gray shadow), the inactive analog of the phospholipase C (PLC) blocker U73122. *B*: summary of data from experiments shown in *A.* Bars in Fig. 2B represent means ± SEM for AngII-evoked Ca^2+^responses before (open bars) during the peak (black bars) and at 60 s after the maximum AngII-elicited calcium response (gray bars). (*; #) *p*<0.05, ** *p*<0.0001; repeated measures ANOVA. n = number of cells from 9 independent experiments.
(TIF)
