# Supplementary figures and images for: Angiotensin-2-Mediated Ca2+ Signaling in the Retinal Pigment Epithelium: Role of Angiotensin-Receptor- Associated-Protein and TRPV2 Channel
Source: PLoS One. 2012 Nov 20;7(11):e49624. doi: 10.1371/journal.pone.0049624 (PMC3502274; doi:10.1371/journal.pone.0049624)

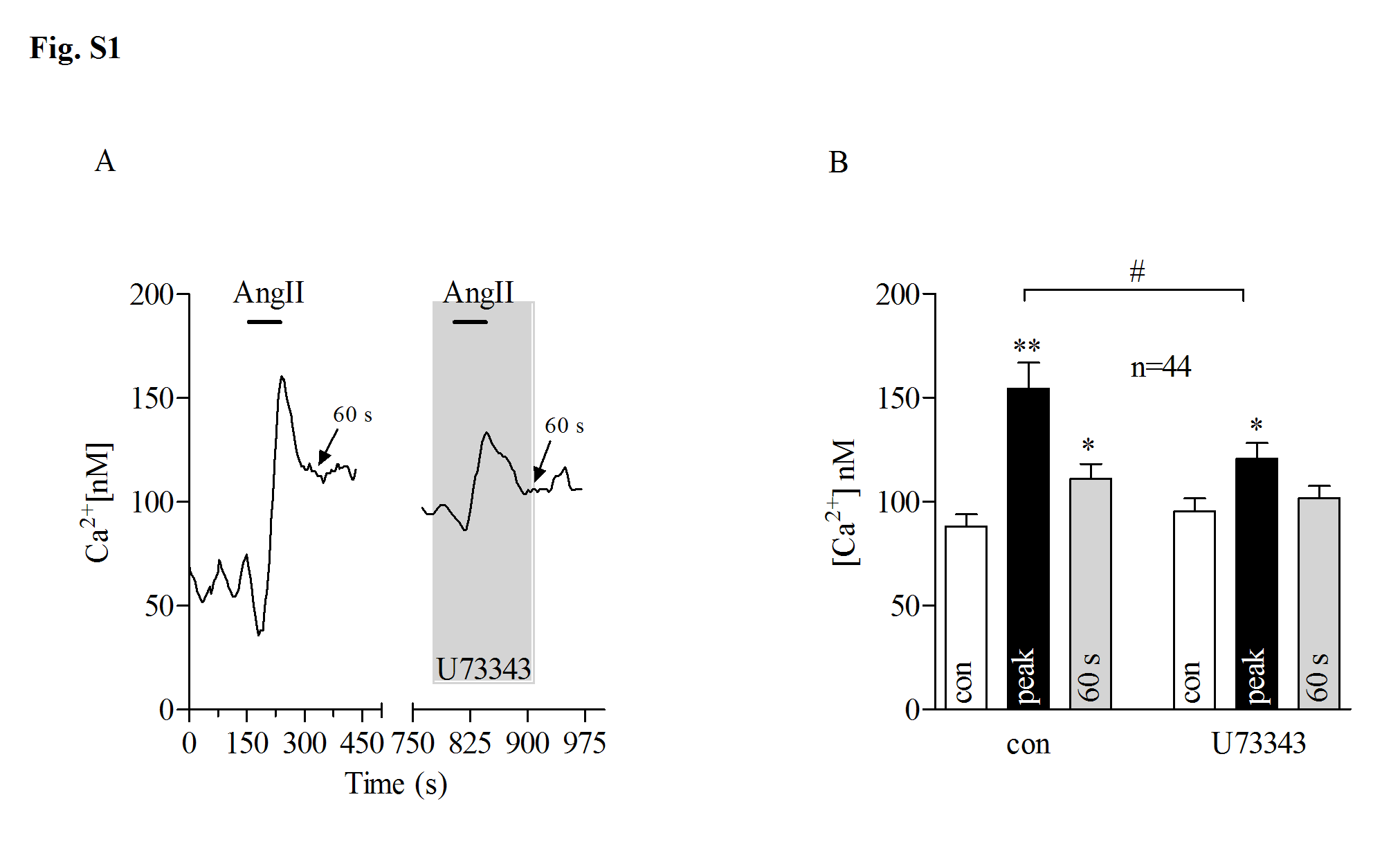

Supplement: Figure S1 — Effect of U73343, the inactive analof of U73122, on AngII-evoked Ca2+response. A: Application of AngII (100 nM) for 80 seconds (bars) caused transient Ca2+response in pRPE cells. Bath application of 100 nM AngII (bars) produced a Ca2+response that was not abolished by co-application of 10 µM U73343 (gray shadow), the inactive analog of the phospholipase C (PLC) blocker U73122. B: summary of data from experiments shown in A. Bars in Fig. 2B represent means ± SEM for AngII-evoked Ca2+responses before (open bars) during the peak (black bars) and at 60 s after the maximum AngII-elicited calcium response (gray bars). (*; #) p<0.05, ** p<0.0001; repeated measures ANOVA. n = number of cells from 9 independent experiments. (TIF) [file pone.0049624.s001.tif]
